# Monthly Summary

**Published:** 1898-10

**Authors:** 


### Monthly Summary.

HOW TO SUCCEED.—Whilst studying chemistry I happened to say to my instructor, " I think." He stopped me instantly. "Think," he said, "what you think does not matter; what do you know? You make an experiment, and get a result; you make another experiment another way, and get the same; you prove your experiments, and then you know. What do you know?" He never had to repeat this; once was enough. What I thought was my own affair; what I knew was the only thing worth consideration, and to know anything worth knowing is not a simple matter.

Take the one subject of filling materials. I worked systematically on these every spare moment, for many years, until my health gave way, and I was compelled to give it up. After all this, what do I know about fillings? Practically nothing, except that I found a more or less satisfactory method of testing results to be expected, and the action of certain metals and alloys in amalgams—merely a surface scratching of what will be got to the bottom of some time, a drop in the ocean of experimental science. Whatever you do, small or great, put all you know into it; take for granted the certain fact that you know very little, find out and prove as much as you can of what you do not know. Above all, keep your eyes open, and do not miss the little chances which often are the beginning of great things. Opportunities come to all, but all do not keep on the watch for them. This is your way up; get out of the crowd at the bottom and build your own steps up, where there is more room, and pleasure, and comfort. It is not enough to do as well as other people—this only leaves you in the outside crowd; you must do better and learn more of something, and you must use your knowledge. A man may be a general failure, but if he learns to do something better than any one else, he may become a great success if he learns to utilize his special knowledge.—Thos. Fletcher, in *Ash's Quarterly*

---

HARDENED AND WASHABLE PLASTER-OF-PARIS.—For the hardening of gypsum, a firm in Heidelberg has taken out a german patent on a process which apparently surpasses all those in existence and furnishes very satisfactory results. Either burnt gypsum is prepared and mixed with the llquid named below, or else the finished articles of hot gypsum, or of mixtures of gypsum and other bodies, are impregnated by painting with the fluid. The same consists of a solution of ammonium triborate in water. For this purpose, boracic acid is dissolved in warm water and a certain amount of ammonia is added, whereby a substance really soluble in water and deviating much in its properties from known compounds results. The saturation of the gypsum, or the painting of the plaster articles, is carried out into the cold. The objects are subsequently rinsed off and dried. The surface becomes very hard after two days and insoluble in water, while the induration in the interior advance more slowly. By means of the fluid described, gypsum floors can be hardened and rendered more durable and impervious to the influences of the weather. Saturated with ammonium borate is said to be especially useful on exterior walls of building, etc. Experiments have proved an antiseptic action of the liquid.—*Scientific American.*

---

Perfect adhesion of gutta-percha to the walls of a cavity may be obtained by wiping out the caxity with copial-ether varnish, or chloroform.

DEATH RATE OF DENTISTS.—Dr. A. B. Freeman, Chicago.—The average age at death of the statesman, clergyman, lawyer, physician and dentist; the farmer, mechanic, laborer, teacher and other vocations seems to vary from sixty-eight years to forty-three years, while the average age at death of the dentist is at forty-five years, and, with a single exception, is the lowest average age at death in any vocation of adult life.

In corresponding with the Superintendent of Vital Statistics for the State of Massachusetts, and with leading actuaries east and west, as well as consulting the United States census reports, I have been unable to verify these statistics; but Dr. Ogle, a celebrated English statistician, in his latest mortality calculations, based on English census returns, states the lowest death rate in any vocation to be that of the clergy—their average age being close to sixty-eight years.

Second he places the legal profession, and includes the statesmen, their average age at death being about sixty-four years.

Farmers and gardeners also die at about this same average age. Physicians come next with a good average of fifty-eight years, while those following occupations requiring cramped, constrained positions, or who are subjected to excessive work, mental or physical, as, for example, silk weavers, lace makers, and, possibly, the shoemaker and dentist, the mortality rate is very much higher.

The deductions to be drawn from such statistics and reports are quite apparent, i. e., that the arduous labor, the severe physical and nervous tension resultant from long operations, often repeated by the busy practitioner, together with the confinement incident to exclusive office life, tend to shorten the years of the dentist's usefulness.

He should ever keep before his mind that first law of nature—self-preservation—and husband all his energies; but how few do this.

At about the time the average dentist has secured his professional education, and by dint of hard work and many sacrifices earned the amount sufficient to repay the obligations incurred during his student life and to properly equip ue office, he is either incapacitated by failing health, or by reason of advancing age, from reaping very much of the fruits from his toil.—*Review.*

---

TO REMOVE ENAMEL PREVIOUS TO CROWNING.—H. H. Johnson, D. D. S.—Take a molar or bicuspid for example, which is to be prepared for an all-gold shell crown. When spacing will be required between the tooth to be crowned and the adjoining tooth, it can be more easily accomplished with vulcan disks, which are thin, strong, flexible and cut rapidly.

Care should be used not to cut the opposite tooth, which can easily be avoided by using pressure toward the tooth to be prepared. By the time sufficient space has been obtained these two sides, the anterior and posterior approximal, have generally been squared up very well, as these thin disks will easily pass down below the festoon of gum and remove the enamel.

The buccal and lingual sides and the approximo-buccal and approximo-lingual corners is generally where the difficulty is.

The buccal and lingual sides down to within a few lines of the gingival margin may be easily and rapidly cut away with small carborundum, which now leaves the crown of the tooth above the neck in a square shape. The disk has flattened it on the approximal sides, and the small wheels on the buccal and lingual sides. By taking small, thin, double convex carborundum wheels these corners may be cut down till the tooth takes a proper shape and the enamel is removed, except a little ring around the neck of the tooth, which generally extends up just beneath the gum margin. To remove this little ring of enamel there is nothing so effective as the vulcan or concavo-convex wheels. The thin edge of these little vulcan cup wheels will pass easily down below the margin of the gum scarcely wounding it and cut off all the irregularities and excrescences. The range of work which these little wheels will do is remarkable; and they are hard and tough and do not wear easily. After cutting and leveling down the bumps, the surfaces of the crown should be planed off smooth by using scalers made very hard and sharp. For this purpose the ordinary diamond pointed, double-edged scalers are best.

By placing the thumb on the crown of the tooth and grasping the scaler firmly in the fingers, commencing at the gum line and pulling toward the cutting edge, the surfaces can be effectually planed off and rendered perfectly smooth. If any enamel should have been left by the wheels it can be easily removed with the scalers in this way.

As these scalers come from the manufacturers, they are too soft for this purpose, and must be hardened. This can be done by heating the point to a white heat and plunging in cold water or oil. Some claim that mercury is still better. —*American Dental Weekly.*

---

Tempering Instruments.—After filing your steel down to the required thickness, polish it, then decide where and how you want to bend it. Heat and bend while it is still hot, using a copper or brass hammer, which will not dent the steel as much as a steel one. Now, after you have it in the required shape, polish out any hammer marks that might be on it, and you are ready to temper. To do this you first coat the instrument with a layer of wet salt and dry it on; this will prevent any scaling, bring your oil or water near to your gas flame. If the instrument is small it is best to hold the burner so that the flame is just over the water, for, if you have the flame away from your cooling fluid, in bringing the instrument to it it loses some of the heat, and your temper will not be as good. Having everything in readiness, heat to a cherry red and suddenly plunge into water. Then polish, being careful not to break the point, which is very brittle; draw the temper by heating quite a distance from the point, using a small flame, watching the colors carefully, so that you will have a straw color at the cutting edge, and blue color at the point where the greatest strain comes; this being a spring temper will lessen the liability of breakage.

When the temper is drawn sufficiently, plunge into cold water and give a final polish, which is easily done by having leather wheels or buffs, five or six inches in diameter mounted on the lathe, using on them a combination of paraffine and emery powder; finish with crocus or rouge. A great deal depends upon this polish, for the finer it is the easier it will be to keep the instrument clean and in an aseptic condition.—*Dr. B. C. Boeseke, in Pacific Medical Dental Gazette.*

---

ONE WAY TO MAKE A GOLD CROWN.—Fit the band to the root of the tooth, contouring as required, and place in position. See where it needs altering to give proper occlusion, and with a half-round file, or other suitable tool, cut away a little more than is necessary to prevent striking against the occluding tooth.

Fit a cap of crown plate to the end and solder. Trim off the edges and replace upon the root, and mark the places where the cusps should be to give proper contour to crown and allow proper occlusion, etc.

With a round, sharp-pointed instrument make some small pits in sheet of asbestos corresponding to the sizes of cusps desired, and with the blow pipe melt the required quantity of gold scrap to form each cusp, and while melted quickly place the handle of the pliers, or anything suitable, on it so as to flatten the top, making small inverted cones.

Place these cones properly upon the cap of crown where cusps are desired, solder to place, and finish as usual.

This way of making a gold crown I found to be the quickest, and gives a more natural contour than any I have tried. It is especially applicable to bicuspids, and gives a greater thickness to the wearing part than the method of fitting to a bite does.—*Dr. L. West, in Items of Interest.*

---

Scratches and stone-marks on mouth mirrors may be removed by polishing them on a felt wheel on the lathe; use plenty of wet pumice and plenty of pressure, but do not allow the mirror to get hot.

FILLING THE UPPER BICUSPIDS.—Dr. G. A. Bowers, Nashua, N. H.—Seventy-five per cent. of the operative procedures are performed on these teeth. Owing to faulty manipulative skill or a lack of conscientious treatment early in life on the part of the dentist, they are either sacrificed or covered with a crown, and generally a gold crown. Frequent examinations, conservative treatment, and conscientious work must be the rule from their eruption. If they are early prone to decay they should be filled with cement or gutta-percha. Watch them closely, renewing the fillings if necessary until the patient is about twenty. At this age if they are of good structure they can be permanently filled, and in doing this an apportunity is afforded the dentist of exhibiting his most artistic skill. When a bicuspid is artistically filled, bringing out the beautiful lines and contour, it is a sure criterion of a good dentist. In the cavity preparation use the chisel freely, never hesitating at sacrificing tooth-structure to accomplish your desired result. Extend the cavity cervically, and beveling so that the gum will come down over the finished filling. The seat of the cavity should be practically flat, with the buccal and palatal grooves extending and rounding into it. A wedge-shaped retention is supplemented in the sulci. Finally, see that your margins are well defined and sharp. A good chisel will do this. Do not use strips or disks, for you will invariably get a rounded edge, and therefore a slivering of the gold at that point. I should always use gold if I could get my pay for it. I think it is perfectly safe and a good practice to use a matrix. I almost, always, however, fill the cervix first burnishing and finishing it. Then apply the matrix and complete the filling. I use Woodward's matrices more than any other, numbers 6, 7, and 8. For special cases where these are not suitable I cut them out of matrix metal and contour them. They can be kept in position with a clamp or orange wood.—*Digest.*

---

An imitator may reap a measure of success, but it will only serve to show how much greater he might have become had he been original.—Items.

MODEL FOR PARTIALS—If it is desired to get an excellent model for an impression in which there are natural teeth, it may be done in this way: Mix the plaster a little thinner than usual, and pour it in at the top of the palate, taking care that the plaster runs down the sides of the teeth and not over them; at the same time the impression must be tapped on the bench or basin in the usual way. After the plaster has covered the whole of the impression, invert it, and by several sharp jerks, throw all the plaster out again into the basin. If the plaster is not too thick this will be readily done, and it will leave the impression with a very thin coating of plaster all over it. If the plaster has not run all over and into every corner, a few taps more will make it do so. Now let the plaster settle to the proper consistency, and fill in the impression in the usual way. When set, the teeth on the model will be found to be perfect and free from bubbles. It will improve the hardness of the plaster if a pinch or two of alum is mixed with it, and it will also hasten its setting. It is also much better for vulcanizing on than a model in which salt has been used.—F. Mackenzie, in *Brit. Jour. Dental Science.*

---

CEMENT.—Of cement, I will say that it has its sphere. It is of especial value in children's teeth, where but little preparation is possible, and in the mouths of delicate people where protracted operations are inadmissible it serves an excellent purpose and lasts well. Cement has the effect of hardening the walls of a cavity, and thus greatly improving the texture of the tooth. Soft teeth should be subjected to such treatment before attempting to fill with gold.—R. W. Meek, in *Pacific Med. Dental Gazette.*

---

The farmer never lived who could reap a crop without sowing seed, and the crop he reaps shows the kind of seed he used and how he used it.—*Items.*

PYORRHEA.—For at least twenty-five years I have treated this trouble almost exclusively with sulfuric acid in its various strengths; not the aromatic, for I do not like its tendency to produce a sediment, nor the different properties due to the spices used. Sulfuric acid and the wine of opium are my chief reliances. I use sulfuric acid up to fifty per cent, and I have no hesitancy in putting that in the pockets, according to circumstances. In some cases it will destroy the soft tissue, and if it does I wait a little longer—from three to six days—before another treatment. I am very successful in mild cases.—Dr. O. E. Hill, in *Cosmos*.

---

MANY have slovenly and annoying habits in conversation that should be mended. A little reflection and observation of our faults will help us much. Hacking and chopping and hesitating are annoying. Repeating a fact or circumstance several times, as though that give it prominence, weakens it. Breaking in on our friends' remarks is rude. Vehemency, demonstration and loud, high-keyed voice are vulgar. To be a good conversationalist is a great accomplishment. The qualities, habits and dominant thoughts—yes, the qualities of the very soul—are manifested by it.—*Items*.

---

RINSING THE MOUTH.—Dr. Crawford says some good things. One which he said a few days since at the Georgia meeting was, that water is the best dental prophylactic. The only trouble is to get people to use it and use it right. Not one in a thousand, he says, knows how to rinse the mouth. Patients should be taught how to rinse the mouth, forcing the water between the teeth, and to do it after eating. Another thing he said that is true. Tooth-brushes as made are too small, the larger a brush the better.—*Dental Weekly*.

---

Listen not to the advice of the man who has failed; follow that of him who has succeeded.—*Items*.

CARIOUS DENTIN.—If I nearly expose a pulp in excavating and there has been no peridental inflammation, I leave a little softened dentin over the pulp and sterilize it. I do not want to kill a pulp that is in a healthy condition. I treat the softened dentin with pure beechwood creosote for a day or two, and then put hydronapthol cement in the bottom of the cavity —one part hydronaphthol powder to two parts of cement powder, mix as any ordinary cement.—Dr. L. G. Noll, in Dental Brief.

---

AMALGAM STAIN.—Some suppose that the stain of an amalgam comes from its oxydation by age. This may add to it, but there is at least nearly as much stain when first made. If the tin is burned it is sure to make black stain. But besides this, and that which cannot be avoided, is the stain of the silver,—*Medico-Dental Gazette.*

---

I have used lately Dr. DeTrey's solila gold, completing the whole filling with hand-pressure, with much satisfaction, but I do not have, as yet, perfect confidence. If this latter stands as well as it looks it will be a great addition to both operator's and patient's comfort, doing away almost entirely with the malleting.—Geo. C. Answorth, in *Digest.*

---

OIL OF CINNAMON.—Inhaling of this causes diminution in number and disappearance of tubercle bacilli from the sputum in phthisis, and tends to cure the disease by arresting growth of bacilli and by preventing organisms capable of from passing along the bronchi to infect new lobules.—*Items*

---

Cement fillings ought to receive a coating of copal-ether before allowing the saliva to get to them.

